# Chronic Polyhydramnios: A Medical Entity Which Could Be a Model of Muscle Development in Decreased Mechanical Loading Condition

**DOI:** 10.3389/fphys.2021.810391

**Published:** 2022-01-13

**Authors:** Slobodan Sekulić, Branislava Jakovljević, Darinka Korovljev, Svetlana Simić, Ivan Čapo, Jelena Podgorac, Ljiljana Martać, Srdjan Kesić, Srdjan Rakić, Branka Petković

**Affiliations:** ^1^Department of Neurology, University Clinical Center of Vojvodina, Novi Sad, Serbia; ^2^Faculty of Medicine of Novi Sad, University of Novi Sad, Novi Sad, Serbia; ^3^Department of Obstetrics, University Clinical Center of Vojvodina, Novi Sad, Serbia; ^4^Applied Bioenergetics Lab, Faculty of Sport and Physical Education, University of Novi Sad, Novi Sad, Serbia; ^5^Department of Neurophysiology, Institute for Biological Research “Siniša Stanković” - National Institute of Republic of Serbia, University of Belgrade, Belgrade, Serbia; ^6^Department of Physics, Faculty of Sciences, University of Novi Sad, Novi Sad, Serbia

**Keywords:** chronic polyhydramnios, fetus, muscle, development, reduced gravity

## Abstract

Polyhydramnios is a condition related to an excessive accumulation of amniotic fluid in the third trimester of pregnancy and it can be acute and chronic depending on the duration. Published data suggest that during muscle development, in the stage of late histochemical differentiation decreased mechanical loading cause decreased expression of myosin heavy chain (MHC) type 1 leading to slow-to-fast transition. In the case of chronic polyhydramnios, histochemical muscle differentiation could be affected as a consequence of permanent decreased physical loading. Most affected would be muscles which are the most active i.e., spine extensor muscles and muscles of legs. Long-lasting decreased mechanical loading on muscle should cause decreased expression of MHC type 1 leading to slow-to-fast transition, decreased number of muscle fiber type I especially in extensor muscles of spine and legs. Additionally, because MHC type 1 is present in all skeletal muscles it could lead to various degrees of hypotrophy depending on constituting a percentage of MHC type 1 in affected muscles. These changes in the case of preexisting muscle disorders have the potential to deteriorate the muscle condition additionally. Given these facts, idiopathic chronic polyhydramnios is a rare opportunity to study the influence of reduced physical loading on muscle development in the human fetus. Also, it could be a medical entity to examine the influence of micro- and hypogravity conditions on the development of the fetal muscular system during the last trimester of gestation.

## Introduction

Microgravity causes a decrease in contractile protein synthesis in skeletal muscles and consequently muscle atrophy, a loss in force and power, and preferential atrophy of antigravity muscles, i.e., extensors over flexors. In slow-twitch antigravity extensor muscles of the astronauts, microgravity induces decreased myosin heavy chain (MHC) I fiber proportion while increasing fast hybrid types (particularly MHC IIa/IIx fibers) (Fitts et al., 2000). Determining the influence of micro- and hypogravity on the human fetus is a necessary condition for colonizing the solar system. Exposing a human fetus to microgravity to assess its impact on muscle development would be unethical. Therefore, it is necessary to establish microgravity analog experimental models. Various ground-based models are used to study the effects of micro- and hypogravity on humans: bed rest studies, parabolic flights, dry and wet immersion. Bed rest studies and immersions cause the same changes in muscles as spaceflight (Watenpaugh, 2016). Bed rest study is not adequate to consider for fetuses, and parabolic flights will expose them only for short time to altered gravitational regime with unknown risk. During intrauterine development, the fetus is in amniotic fluid and wet immersion could provide a clue for the model of the influence of altered gravitational regime on muscle development in the human fetus.

## Physical Condition in the Second Half of Gestation

During the second half of gestation, the specific weight of the fetus is 1,040–1,050 g (Lu and Hsu, 1999) and the specific weight of the amniotic fluid is 1,002–1,010 g (Okai, 1986). The surface tension of the amniotic fluid is 14–58 dyn/cm (Muller-Tyl and Lempert, 1975), allowing contact between the fetal skin and the walls of the intrauterine cavity. Intrauterine pressure of amniotic fluid represents the sum of air pressure in the atmosphere, elasticity of the walls of amniotic fluid and hydrostatic pressure produced by the amniotic fluid column height. *In vivo* measurements showed that the pressure is 0.66 kPa (Sideris and Nicolaides, 1990). This value represents the difference between the outside air pressure in the atmosphere which on the sea level is 101,325 kPa and the intrauterine pressure which is higher by 0.66 kPa. Mechanical stress the muscles are exposed to is the result of the actions of: kinetic energy of the fetal muscles, potential energy of the fetal muscles reduced to 60–80% by comparison with extrauterine conditions, deformation of the amnion and uterus wall, overcoming the resistance due to viscosity of water (Sideris and Nicolaides, 1990; Young and Freedman, 2016; Verbruggen et al., 2018). In order to deform the walls of the intrauterine cavity, fetus must overcome the elastic forces of uterus and amnion. Amniotic fluid affects the decrease of the potential energy necessary for the fetus movement through buoyant forces (Young and Freedman, 2016). Until the 24th week of gestation, the fetus is smaller than the intrauterine cavity, it is in a condition similar to neutral buoyancy and weighs 10% of its actual weight. During the last trimester of gestation, the fetus outgrows the intrauterine cavity, the buoyant forces are less expressed, and it weighs 60–80% of its actual weight (Sekulic et al., 2005). Potential energy generated by the fetal muscles is decreased by 20–40% because of this compared to the fetus being outside of the influence of the buoyant forces.

## Intrauterine Physical Condition in Microgravity

Hydrostatic pressure is absent in microgravity. Intrauterine pressure is equally distributed in amniotic fluid. Buoyant forces are present, but they are pointed in all directions to an object immersed in a fluid with sum value zero. Mechanical stress the muscles are exposed to is the result of the actions of: kinetic energy from the muscular activity, deformation of the amnion and uterus wall, overcoming the resistance due to viscosity of water. Potential energy of muscle movements is absent. Mechanical stress against the muscles is reduced by the value of the potential energy in comparison to the movements in the 1G environment (Young and Freedman, 2016).

## Amniotic Fluid Production and Resorption

During the last trimester of gestation, fetal urine and fetal lung liquid represent the source of amniotic fluid. Fetal urine output increases from 110 mL/kg fetal weight/24 h during the 25th week up to approximately 200 mL/kg fetal weight/24 h at term (Rabinowitz et al., 1989). Based on some animal studies, the fetal lungs excreted about 60–100 mL/kg fetal weight/24 h (Gilbert and Brace, 1993). The fetal swallowing decreases the amniotic fluid for approximately 200–250 mL/kg fetal weight/24 h (Abramovich et al., 1979). Experimental data on sheep showed that absorption of amniotic fluid from the intramembranous pathway is in the range of 200–400 ml/day (Wintour and Shandley, 1993). Intramembranous pathways are regulated by: 1. Vascular endothelial growth factor (Matsumoto et al., 2001), 2. Transmembrane water channels (aquaporins 1, 8, 9), 3. Inhibitors of absorption in fetal urine, and 4. Intra-amniotic prostaglandin E2 (Brace and Wolf, 1989; Beall et al., 2007).

## Chronic Polyhydramnios and Physical Intrauterine Condition

Chronic polyhydramnios represents a condition with an increase of the amniotic fluid during the last trimester of gestation. It increases the buoyant force and decreases the mechanical stress to the fetal musculoskeletal system, and could reduce the apparent weight of the fetus from physiological values of 60–80% to 10–15% of its actual weight (Sekulic et al., 2010). In polyhydramnios, hydrostatic pressure rises up to 2.8 kPa (Bergh et al., 2019). This slight increase in hydrostatic pressure does not increase the viscosity of water. Water is incompressible. In polyhydramnios, the potential energy necessary for the fetal movements is reduced by 10–15% in comparison to extrauterine conditions (Sekulic et al., 2010). Additionally, polyhydramnios distends the wall of the intrauterine cavity, providing more space for fetal movements. In this way, fetal movements are less likely to deform the wall of the intrauterine cavity. Mechanical stress the muscles are exposed to is the result of the actions of: kinetic energy from the muscular activity, 10–15% of the muscle potential energy compared to extrauterine conditions, variable reduced deformation of amnion and uterus wall compared to physiological gestation, overcoming the resistance due to viscosity of water. Sum of the mechanical stress on fetal muscles compared to physiological pregnancy is reduced (Sideris and Nicolaides, 1990; Young and Freedman, 2016; Verbruggen et al., 2018). It is necessary to develop a biophysical model to calculate the sum of mechanical forces for fetal movements in microgravity and polyhydramnios. Decreased buoyant forces with reduced force to deform the wall of the amniotic cavity offer the possibility to calculate the same value of the total mechanical stress which the fetus is exposed to in microgravity and polyhydramnios. In the available literature there are physical models that describe the mechanical stress the fetus is exposed to in the physiological conditions. Stresses and strains on the human fetal musculoskeletal system were determined based on the force of fetal movements necessary to deform the uterine wall during the second half of gestation. Kick reaction force increases from 28.85 ± 1.88 N during the 20th gestation week to the maximum value of 46.64 ± 5.30 N during the 30th gestation week (Verbruggen et al., 2018). The maximum principal stress on the femur and tibia rises from the 20th until the 35th week of gestation from approximately 15–80 kPa. During the same period, the maximum as well as the minimum principal strain rise for femur from 0.1–0.2 to 1.2%, and tibia approximately from 0.1 to 0.6% (Verbruggen et al., 2018).

## Etiology of Polyhydramnios

The incidence of polyhydramnios is approximately 1% in human gestations (Hill et al., 1987). Incidence of the acute polyhydramnios is 1 case in 2,685 deliveries, while the chronic polyhydramnios appears in 1 of 102 pregnancies (Desmedt et al., 1990). The cause of polyhydramnios could be a disturbance of any of the mentioned factors in the production and resorption of the amniotic fluid. The current diagnostic capabilities do not allow the exact *in vivo* determination of the factor that causes polyhydramnios, but for some diseases, it is possible to comprehend the pathophysiological mechanisms. Impaired swallowing as a consequence of muscular disorders, as well esophageal or duodenal atresia can prevent absorption of the amniotic fluid (Hamza et al., 2013). The increased cardiac output associated with fetal anemia (for example Rh isoimmunization) can cause an increased urine production and, consequently, an increased volume of the amniotic fluid (Hamza et al., 2013). The increased volume of amniotic fluid could be the consequence of transudation of fluid across the fetal meninges in case of the central nervous system malformations (encephalocoele, anencephalus) or a lack of antidiuretic hormone and the resultant polyuria (Wallenburg and Wladimiroff, 1977). Maternal gestational diabetes mellitus accompanied by fetal hyperglycemia could cause an increased osmotic diuresis which subsequently leads to polyuria and polyhydramnios (Hamza et al., 2013).

In the majority of cases, it is not possible to find an underlying disease as a cause of polyhydramnios. 50–70% of cases are classified as idiopathic form (Dorleijn et al., 2009). In mild cases, diseases are present at 17%, while in moderate to severe polyhydramnios the percentage of underlying diseases increases up to 91% (Hill et al., 1987).

## Determination of Myofiber Types During Development

Muscle fibers containing the MHC type I had a 10-fold lower unloaded shortening velocity, maximum power output, constant rate of tension rising, and a 3-fold lower ATPase and tension cost than the MHC 2X fibers. The MHC 2A type is intermediate. Type I muscle fibers had a relatively poorly developed sarcoplazmatic reticulum contrary to the muscle fiber type II. MHC type 1 constitutes the basis for the muscle fiber type I (slow-twitch fibers), while the MHC 2A and 2X are the basis for the muscle fiber type II, fast-twitch fibers (Canepari et al., 2010).

The determination of myofiber types is caused by the expression of MHC genes. In the model of altricial species (rat, mice), it is shown that the expression of MHC genes in adult individuals depends on the intrinsic factors (muscle cell lineage). It also depends on the extrinsic factors: innervation, mechanical loading (contractile activity, muscle stretch), and thyroid hormones. Reduced neuromuscular activity, hyperthyroidism, and decreased mechanical loading stimulate slow-to-fast fiber transition. In the opposite case, increased neuromuscular activity, hypothyroidism, and increased mechanical loading lead to fast-to-slow fiber type transition (Soukup and Smerdu, 2015).

Expression of genes responsible for the fast-twitch isoforms is evident after the 14th week of gestation in humans. During the 24th week of gestation, human hind limb muscles have the fast contractile protein isoform phenotype (Sutherland et al., 1991). In the period from the 20th to the 24th week, approximately 5–10% of the fibers correspond to type I muscle fibers. The remaining 90–95% of the fibers represent the type II muscle fibers. Between the 24th and the 34th week of gestation, the predominant change is a modest increase in the size of the type II muscle fibers. During the late histochemical differentiation, from the 34th to the 38th week of gestation, small type I muscle fibers appear in large numbers (Volpe, 1987; Romero et al., 2013).

## Muscle Types Development and Mechanical Loading

Data about muscle development in the case of polyhydramnios are missing either for humans or for experimental animals. Chronic polyhydramnios could be a model of decreased mechanical loading in the period of gestation when buoyant force has less influence on the fetus. Based on previously presented data this happens after 24th weeks of gestation. In this last trimester of gestation muscles of human fetuses are in the phase of histochemical differentiation with both types of MHC fibers present (Volpe, 1987; Sutherland et al., 1991; Romero et al., 2013). The possible influence of polyhydramnios could be extrapolated based on data related to the influence of decreased mechanical loading during the stage of muscle development when both types of MHC fibers are present.

Physiologically human fetus assumed flexed posture during the last trimester of gestation. Additionally, the fetus was overgrowth intrauterine cavity, and to move in intrauterine space it must use extensor muscles of the lower extremities and spine to overcome the tension of the intrauterine wall. The soleus muscle is often used as representative of antigravity extensor muscle. As models of decreased mechanical loading during the stage of differentiation of muscle fiber types, we presented published data about soleus muscle of sheep and rats. A change of fetal and amniotic fluid volume ratio in sheep and rats during the last periods of gestation is similar to that in humans. What is different is the stage of muscle development during this period. Between the 83rd and 100th day of gestation in sheep (duration of gestation 146 days), there is a dramatic shift in the fetus/amniotic fluid volume ratio from 1:2 to 2:1 in favor of the fetus. At the end of gestation, the ratio is 11:1 (Cloete, 1939). In the rat (duration of gestation 21–23 days) on the 11th day of gestation ratio of the volume of fetus/amniotic fluid is 1:5, on the 16th day 1:1, on the 18th day 2.5:1, and on the 20th day 7:1 (Park and Shepard, 1994). Period of decreased buoyant force for humans, sheep, and rats could last around 1/4 of gestation.

Sheep belongs to precocial mammal species and has prenatal differentiation of the MHC to type I and II. Consequently, muscles attained slow and fast-twitch profiles during intrauterine development. The soleus muscle consisted of type I fibers already on the 90th day of gestation. As gestation advances, the soleus muscle contracted more slowly, and on the 140th day, it lasted 111.9 ± 6.6 ms. There was no significant change with gestational age in the proportion of type I and type II fibers in two fast-twitch muscles, medial gastrocnemius, and flexor digitorum longus. Duration of contraction for those two muscles remains unchanged after the 125th day of gestation and is 41–44 ms (Javen et al., 1996).

In sheep fetuses, intrauterine development of the muscle phenotype is impeded in the absence of the physical support normally provided by the tibial bone. The contractile and activation profiles of the soleus muscles were significantly affected by tibial bone resection in sheep fetuses on the 90th day of gestation (both types of extrafusal muscle fibers are present at that time). The experiment shows a decrease in the time to peak of the twitch responses from 106 ± 10.7 ms to 65.1 ± 2.48 ms, fatigue profiles more characteristic of those observed in the fast-twitch muscles, and Ca^2+^ and Sr^2+^ activation profiles of the skinned fibers similar to those from intact hind limbs during the earlier stages of gestation. The changes suggest a transition toward the fast-twitch muscle fiber (West et al., 2000).

In the rat, which is altricial species, intrauterine muscle differentiation is lagging behind humans as well as sheep. From the 19th embryonic day until the end of gestation on the 22nd day a strong bias toward fast isoform expression is evident for all gene families (Sutherland et al., 1991). In the soleus muscle differentiation is barely evident at birth. In the 1-day-old newborn, 60–80% of the fibers were in the myotube stage of development. In the soleus muscle, on the 4th day of gestation the percentage of fibers expressing type I was 55% while for type II was 41% (Kawano et al., 2010). During the next 2 months, differentiation proceeds rapidly (Curless and Nelson, 1976). During postnatal muscle development in the case of tail suspension, the experimental group has a significantly lower content of MHC type I in the soleus muscle compared to the control group 53 vs. 82% (Kawano et al., 2010).

It is shown based on presented data that regarding types of MHC muscle fibers, we could expect transition toward the fast-twitch profile in case of chronic polyhydramnios in human pregnancies. Although MHC expression is mainly postnatal in rats and prenatal in sheep, continuity of gravity influence on muscle development during prenatal and postnatal development exists.

## Prenatal Muscle Hypotrophy—Atrophy in Case of Decreased Loading

Absent fetal movements leads to the condition Fetal Akinesia Deformation Sequence. This syndrome is characterized by: skeletal muscle hypotrophy-atrophy, long bone hypoplasia, arthrogryposis multiplex congenital (all three because of absent extremities movements), pulmonary hypoplasia (because of absent breathing movements), high arched hard palate (because of absent negative pressure produced by tongue movements) (Adam et al., 2018). In line with this, in addition to the expected transition toward the fast-twitch profile in the case of chronic polyhydramnios, hypotrophy-atrophy of skeletal musculature is also to be expected. Most affected would be muscles which are the most active. The most frequent movements involve the extension of the spine and movements of the legs during the second half of gestation (Kozuma et al., 1997). Because of this most affected will be muscles responsible for the extension of spine and leg muscles.

## Experimental Models of Polyhydramnios in Animals

While research in medicine could be conducted without invasive procedures with indirect inference based on electromyographical characteristics or postmortem analysis, experimental animals could allow invasive procedures. In sheep, it is possible to induce polyhydramnios by chronic intravenous infusion of angiotensin I [182 μg/(kg/day)] into fetal lambs (Anderson and Faber, 1989). Transgenic aquaporin 1 (AQP-1) knockout mice fetuses produce a greater dilute amniotic fluid volume (Mann et al., 2005). Data regarding muscle development in these experimental models are absent and further research should give a better insight into this topic.

## Final Remarks

It should be noted that the intrauterine period is a critical period for muscle fibers. Their definitive number is achieved before birth (Sutherland et al., 1991). Whether polyhydramnios causes long-lasting qualitative changes, such as muscle fiber type transition/determination, or/and quantitative changes, such as muscle hypotrophy, further investigation should show. Also, these changes in case of a preexisting muscle disorder have the potential to deteriorate the muscle condition additionally. Idiopathic chronic polyhydramnios is a rare opportunity to study the influence of reduced physical loading on muscle development in the human fetus and together with changes on fetal bones (Sekulic et al., 2010; Sekulic and Petkovic, 2019), it could be a medical entity which provide data about possible influence of micro- and hypogravity conditions on the development of the fetal musculoskeletal system.

## Author Contributions

SSe contributed to the conception and design of the article and interpreted the relevant literature. BJ, DK, SSi, IČ, JP, LM, SK, SR, and BP critically revised the manuscript. All authors approved the final version of the manuscript.

## Conflict of Interest

The authors declare that the research was conducted in the absence of any commercial or financial relationships that could be construed as a potential conflict of interest.

## Publisher’s Note

All claims expressed in this article are solely those of the authors and do not necessarily represent those of their affiliated organizations, or those of the publisher, the editors and the reviewers. Any product that may be evaluated in this article, or claim that may be made by its manufacturer, is not guaranteed or endorsed by the publisher.
